# Atrial Tachycardia Masquerading As Inappropriate Sinus Tachycardia (IST) After COVID-19 Infection: A Matter of Concern?

**DOI:** 10.7759/cureus.20090

**Published:** 2021-12-01

**Authors:** Anamika Giri, Dhruv Talwar, Sourya Acharya, Daljeet K Saggu, Sunil Kumar

**Affiliations:** 1 Department of Medicine, Jawaharlal Nehru Medical College, Datta Meghe Institute of Medical Science (Deemed to be university), Wardha, IND; 2 Department of Medicine, Jawaharlal Nehru Medical College, Datta Meghe Institute of Medical Sciences (Deemed to be university), Wardha, IND; 3 Department of Cardiac Electrophysiology, Asian Institute of Gastroenterology, Hyderabad, IND

**Keywords:** post-covid sequelae, long covid, radiofrequency ablation, atrial tachycardia, covid-19

## Abstract

COVID-19 Infection has wrecked havoc all over the world; the spectrum of this disease ranges from asymptomatic mild cases to severe cases such as acute respiratory distress syndrome (ARDS). Not only the acute infection but post COVID sequelae are also a cause of concern. Post-COVID states or Long COVID are the sequences of complications following the active infection. As post COVID sequelae are unpredictable it is absolutely the need of the hour to educate physicians and make them aware of all possibilities. We report one such case of a post COVID recovered young lady, who presented with drug-refractory recurrent palpitations. She was initially suspected to have inappropriate sinus tachycardia. But electrophysiological study confirmed the diagnosis of atrial tachycardia which was successfully ablated. The patient now has completed six months of follow-up and is off any medication.

## Introduction

SARS‐CoV‐2 infection is associated with innumerable inflammatory markers that are thought to play a poignant role in the etiopathogenesis of cardiogenic and varied arrhythmia-associated complications. Palpitations are not an uncommon concern in the primary care setting and sinus arrhythmia is the most reported during active infection as well as post-COVID sequelae [1]. Despite the fact that clinical symptoms of COVID-19 are primarily respiratory, there is a rising number of infectious patients suffering from serious cardiological complications as well as other systemic complications are seen to be documented in a significant number of patients with COVID‐19 [2,3].

## Case presentation

A 24-year-old female with SARS-CoV-2 infection approximately one month back visited the hospital with complaints of palpitations, episodic dizziness and mild dyspnea. These complaints were sudden in onset and gradually progressed. Palpitations were more pronounced on getting up in the morning and breathlessness was present at rest. These complaints were aggravated on exertion.

On clinical examination, the patient had tachycardia pulse 120/min, regular at rest which increased to 138/min after standing and 160 beats/min with mild exertion (three-minute walk test) she started to complain of mild dyspnea. Throughout her saturation (SpO_2_) on room air remained 98%. Apart from tachycardia, her complete systemic examination was normal. Blood pressure in right arm supine position was 116/72 mm of Hg and after three minutes of standing it was 92/60 mm of (a postural fall of (24/12 mm of Hg). However, she did not complain of presyncope symptoms.

On systemic examination, the patient was conscious and well oriented to time, place and person. Respiratory system examination was normal. The cardiovascular system revealed normal heart sounds. There were no murmurs, S3/S4. The abdomen examination was normal.

**Figure 1:**
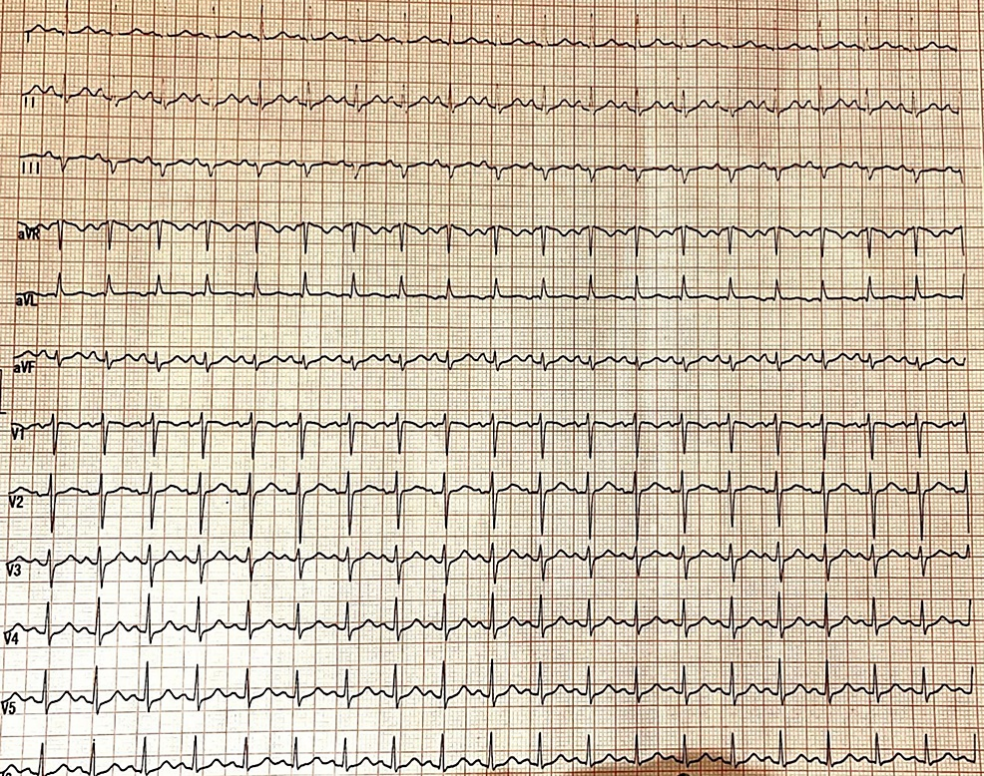
ECG showing sinus tachycardia

Laboratory investigations are mentioned in Table 1. Kidney and liver function tests were normal. Serum CPK-MB, cardiac-specific troponin I and NT-proBNP were within normal limits. 2D echocardiogram was normal. Thyroid profile was normal. ECG revealed sinus tachycardia with a normal QTc (Figure 1). The patient was advised high-resolution computed tomography (HRCT) of Thorax which showed no focal lesions in bilateral lung fields, no significant mediastinal lymphadenopathy, no obvious pleuro-parenchymal abnormality.

**Table 1:** Laboratory investigations of the case

Components	Values
Hemoglobin	12.1 g/dL
Red Blood Cells	4.35 million/mm^3^
White Blood Cells	11,800/mm^3^
Platelets	363,000/mm^3^
Erythrocyte Sedimentation Rate	32 mm/hour
C-Reactive Protein	0.5 mg/L
D-Dimer	0.69 mcg/microliter
Random Blood Sugar	128 mg%

Contrast-enhanced CT (CECT) thorax with pulmonary angiography was advised in view of slightly raised D-Dimer and it was normal which ruled out pulmonary embolism. The patient was started on tab ivabradine 5mg BD and tab metoprolol 12.5mg OD after which the patient’s symptoms started improving.

**Figure 2:**
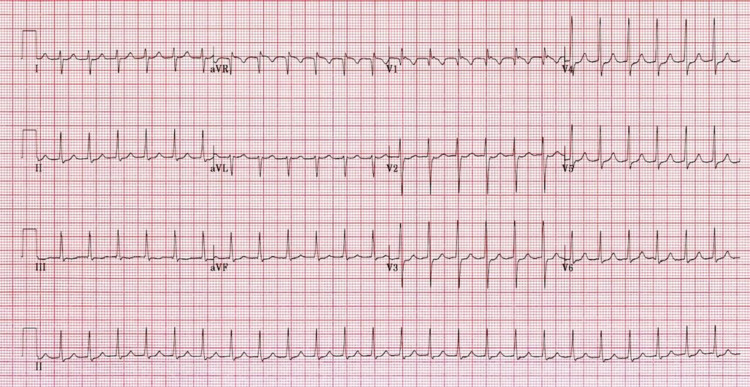
ECG showing supraventricular tachycardia

Even after drug therapy, the patient complained of persistent tachycardia, pulse rate of 140/min even at rest. The patient’s medication doses were stepped up to tab ivabradine 7.5mg BD and tab metoprolol 50mg BD despite this patient complained of persistent episodes of palpitations along with mild breathlessness and occasional chest pain. The patient’s 48 hours Holter monitoring was done, which showed no obvious abnormalities. Two months later the patient had an episode of dizziness after which ECG was taken immediately which showed supraventricular tachycardia (Figure 2), which terminated spontaneously after vagal maneuvers. The supraventricular tachycardia was thought to be an atrioventricular nodal reentry tachycardia (AVNRT) after which the patient was started on tab dilzem SR 90mg OD and all other medications were stopped.

**Figure 3:**
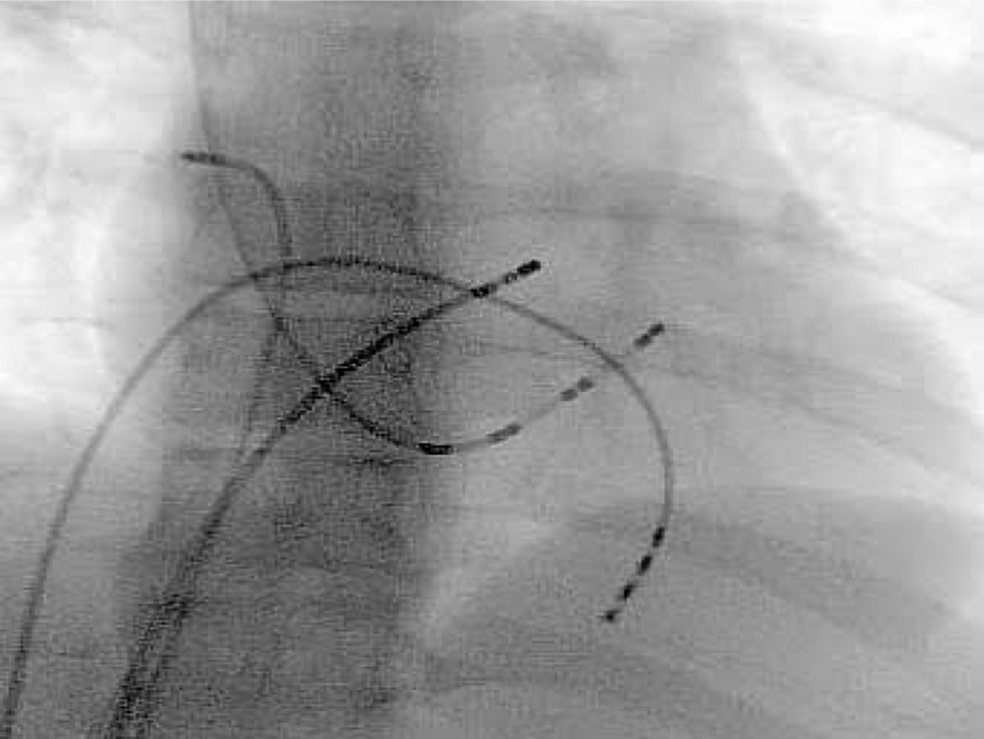
Fluoroscope image of radiofrequency ablation

**Figure 4:**
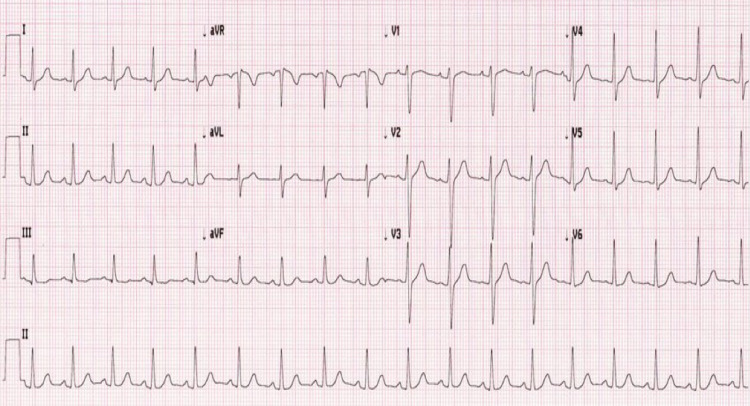
Post-ablation ECG showing sinus rhythm

The patient was advised electrophysiological study and radiofrequency ablation in view of her complaints. An electrophysiological study was done and mapping showed atrial reentrant tachycardia originating from mid-high crista. Radiofrequency ablation of the mid-high crista was done (Figure 3). The procedure was uneventful. The patient was put on tab propranolol 20mg BD for three months along with tab aspirin 75mg OD for one month. Post-procedure, the patient’s tachycardia settled down and the patient was eventually weaned off medications after three months of improvement of the symptoms pulse rate of less than 90/min (Figure 4).

## Discussion

Presentation of patients suffering from COVID-19 falls on a varied spectrum [4,5]. Furthermore, there are reports showing patients suffering from cardiac dysfunction post-COVID as well as ventricular fibrillation or cardiac catastrophe such as pulseless activity during the recovery phase of this deadly disease [6]. This can be attributed to circulating low oxygen and electrolyte disturbances, which could lead to episodic arrhythmias, or central nervous system disturbances caused by SARS-CoV-2 disease. It has been hypothesized that COVID-19 infection affects the autonomic nervous system. The relationship between the two is complex: the well-documented cytokine response storm of COVID-19 results from sympathetic activation inducing proinflammatory cytokine release [7,8]. Conversely, vagal stimulation results in anti-inflammatory responses, suggesting possible therapeutic targets in the autonomic nervous system [9].

Inappropriate sinus tachycardia (IST) can be defined as chronic nonparoxysmal sinus tachycardia, which is an uncommon disorder that affects patients with no evident cardiac disease or other causes of tachycardia, such as anemia, fever or hyperthyroidism and is usually treated as an exclusionary diagnosis [10]. The pathogenesis of IST is uncertain; however, it is thought to entail intrinsic nodal hyperactivity in conjunction with autonomic hyperactivity mediated by neurohormonal effects. Because of the syndrome's obscurity and the complexity of its nature, managing patients with IST remains a struggle. In IST, there is no single treatment that can effectively lower the rate and relieve symptoms.

Arrhythmias, involving both atrial and as well ventricular, have been witnessed in COVID-19 patients who had never had arrhythmia before. Individuals with increased troponin T levels had an increased rate of fatal arrhythmias, such as ventricular tachycardia and or ventricular fibrillation, than patients with baseline troponin T levels. COVID-19 does not cause arrhythmias or conduction system disease early or frequently, and the bulk of symptoms are connected to respiratory system involvement. While sinus tachycardia is commonly associated with the physiologic response to viral infection, arrhythmias other than sinus tachycardia have been recorded at a high rate.

Arrhythmias are a common side effect of infections, and it appears that viral myocarditis, which affects the heart's conduction system, is the most common cause. SARS and MERS, which are closely related to the COVID-19 virus, have been shown to produce arrhythmias such as sinus bradycardia and tachycardia [11,12]. Furthermore, new-onset arrhythmia increased biomarkers and atrial arrhythmias were more prevalent in COVID-19. Arrhythmias have been linked to viral infections that cause viral myocarditis in the past, and substantial data shows that this may be the case in COVID-19-infected patients as well [13].

Arrhythmias were prevalent and linked with high morbidity and mortality in a large cohort of COVID-19 patients hospitalized around the world. Atrial arrhythmias were the most prevalent, accounting for 80% of all arrhythmia cases. Despite the fact that most patients with cardiac arrhythmias had no prior history of arrhythmia, they had a significant burden of medical comorbidities. Patients were frequently extremely ill and had a high death rate, with just half of those admitted surviving to release [7].

## Conclusions

Currently, there is little information about neuroinvasive potential of SARS-CoV-2 and resultant autonomic dysfunction. Better knowledge is essential for determining whether continued arrhythmia monitoring is required during hospitalization and after discharge. Understanding these dangers associated with COVID-19 disease will aid in the tight monitoring of affected patients as well as the advancement of information about such problems for global public health.

There are currently no extensive primary analyses on arrhythmias and probable mechanisms in the literature. This makes distinguishing between arrhythmias produced by hypoxia, metabolic problems, inflammatory syndrome, comorbidities, and drugs against direct viral effects on the heart challenging. Further research is needed to thoroughly demonstrate this association and assess long-term repercussions.
